# “Shape is everything: on proteins’ functions”: Author

**DOI:** 10.1007/s10539-025-10005-5

**Published:** 2025-12-11

**Authors:** Francesca Bellazzi

**Affiliations:** Department of Philosophy, Leverhulme Trust Early Career Fellow, Durham University, 50 Old Elvet, Durham, DH1 3HN UK

**Keywords:** Unctions, Proteins, Structure, Biochemical kinds

## Abstract

Proteins are often defined as the molecules that enable life thanks to the special functions they display. But what are proteins’ functions? Despite their relevance in various debates, the answer to this question is often left implicit. This paper argues that a correct characterisation of proteins’ functions must consider the native structure of the protein, building on Bellazzi’s definition of biochemical functions: proteins’ functions are dispositions associated to specific chemical and geometrical structural properties relevant for the tertiary and quaternary structure of proteins, and they contribute to specific evolved biological processes. Section 1 introduces the problem of protein’s functions. Section 2 unpacks the “structure problem” and the “function problem”, where the former inquiries into the function bearer of proteins’ function and the second into the relevant account of function. Section 3 presents an answer to the structure problem by arguing that the focus should be on native structure. Section 4 and Sect. 5 focus on the function problem and the account defended. Section 5 also considers two specific case studies, haemoglobin and crystallins, and a critical evaluation of the account. Section 6 concludes.

## Introduction

Proteins are some of the molecules that make life possible, being its building blocks, motors and regulators thanks to the unique combination of structural and functional properties they display. Their centrality to life is reflected in the etymology of the term itself, derived from the Ancient Greek *proteios*, meaning “primary”. Proteins are uniquely insightful for scientific research, as evidenced by the many prestigious awards recognizing advances in this field,^1^ and for philosophical investigation, lying at the intersection of biology and chemistry.

Disciplines such as biochemistry, molecular biology, and structural biology devote significant attention to understanding the relationship between a protein’s structure and its function and the concepts of biological function and chemical structure have been examined in philosophical literature (see, for instance, Garson 2016 and Hendry 2023 respectively). Moreover, there has also been a more general growing interest in the philosophical features of biochemical kinds, reinforcing the need to explore how structure relate to function in proteins (Slater 2009; Tobin 2010; Goodwin 2011; Bartol 2016; Havstad 2021; Tahko 2020; Bellazzi 2025). While some recent works apply existing accounts of biological function to molecular case studies (e.g., Bellazzi 2025; Havstad and Palazzo 2022; Dupin 2023), the question remains: what exactly are proteins’ functions?^2^ This paper will consider this question by critically engaging with current accounts of function, giving particular attention to the role of a protein’s three-dimensional structure in function attribution.

Providing a clear account of protein function is far from straightforward—an issue common to the attribution of function to molecules more generally (Germain et al. 2014; Ratti and Germain 2022; Havstad and Palazzo 2022; Dupin 2023). This is so because multiple factors are relevant for proteins’ function, such as structural features, evolutionary history, and environmental context. Existing literature has taken divergent approaches: some identify proteins’ functions by looking at evolutionary features (as Bartol 2016), and others reduce proteins’ functionality^3^ to structural features and the amino acids’ chains composing the proteins (as Goodwin 2011; Tahko 2020). This affects the classification and kindhood of proteins, as classification systems aim to integrate structural, functional, and evolutionary dimensions (Slater 2009; Tobin 2010; Havstad 2018, 2021).

This paper provides the starting point for an account of proteins functions’ and argues for the “shape is everything” thesis. Proteins’ functions are dispositions associated to specific chemical and geometrical structural properties relevant for the native structure of proteins, and they contribute to specific evolved biological processes. This account is based on two partial claims, discussed as the “structure” and the “function” problem. The first asks which aspects of protein structure are relevant for their function. The second asks what notion of function can be applied to proteins. The answer to the structure problem is given by the focus on the three-dimensional structure of proteins at their native state. The answer to the function problem is given by a modified version of the biochemical view of functions I previously proposed, where biochemical functions are associated to indirectly selected chemical functional groups (Bellazzi 2023, 2025). Differently from this definition of biochemical function, the proposed account underlines the role of the three-dimensional shape of the proteins rather than considering only the chemical functional groups. Its advantage is that by focusing on the folded structure it allows for a characterisation of proteins’ functions consistent with contemporary scientific research and for an account that goes beyond the presence of functional groups. This also stands against taking a reductionist view for which the primary structure of proteins delivers all the relevant properties for proteins characterisation (*contra* Goodwin 2011, Tahko 2020).

Together with providing a useful characterisation of proteins’ functions, this paper contributes to different debates. First, it adds to the broader biological function literature, including the causal-contribution account (Cummins 1975, Craver 2001), the selected-effect theory of functions (Neander 1983, 1991; Christie et al. 2022; Garson 2019; 2023; Fagerberg and Garson 2024) and the organisational view of function (Saborido et al. 2011). It does so by explicitly highlighting the role that structural features play in determining functions. This can also be extended to the morphology of traits and its impact on function, a relationship extensively studied in structural morphology. While the importance of structure for function is often acknowledged, it remains largely implicit in philosophical discussions, except, notably, in debates between structuralism and functionalism in evolutionary developmental biology (Novik 2023). The case of proteins offers a clear example where structure–function relationships are particularly evident, providing an opportunity to bridge molecular and morphological accounts of functionality. This supports a more unified view in which structure and function can be considered two sides of the same coin. Second, this paper contributes to the debates on the differences and similarities between chemical and biological entities. Functions are easily attributed to living things but many resist the import of an historical or functional analysis to physical or and chemical entities. The distinction between the chemical-physical and the biological is often treated as fundamental and can impact the characterisation of life (Germain et al. 2014; Ratti and Germain 2022). For instance, Garson (2023) suggests that the *functional mechanisms* used in biology and those in physics and chemistry are of *different kinds*, to the extent that we can’t import the terminology from one to the other. This might seem straightforward when considering atomic structure in isolation or chemical elements such as gold. Chemical functional groups as reactivity profiles of molecules can be considered only analogous to the function of the heart to pump blood (Dupin 2023). However, things get less neat when we consider entities like proteins which are chemical macromolecules (and thus excluded from “real functions”) that play fundamental functions in biological organisms. Exploring proteins’ functions can thus have a broader impact on the notion of functions in life and non-life. Thirdly, an account of proteins’ functions allows for a better understanding of the kindhood and classification of proteins, as a clearer notion of functionality can improve the debates between pluralism (Slater 2009), functionalism (Tobin 2010; Bellazzi 2025), reductionism (Goodwin 2011; Tahko 2020), and dualism (Bartol 2016) regarding biochemical kinds. Thanks to a defined notion of proteins’ functions, these debates could become more straightforward.

The structure of the paper is the following. Sect. "Where is the problem with proteins functions?" introduces the problem of proteins’ functions in terms of a two-fold problem, the “structure problem” and the “function problem”. Sect. "Shape matters for function" focuses on the structure problem and illustrates that the properties of the native folded state of the protein are those relevant for functionality. Sect. "Unpacking the function problem" examines the causal contribution, selected effect and biochemical view of function and why they might not be suited to for a specific characterisation of proteins’ functions. Sect. "Shape is everything" develops the “shape is everything” thesis, building on my first characterisation of biochemical functions (Bellazzi 2025). It argues that proteins’ functions are dispositions associated to specific chemical and geometrical structural properties of folded shape of proteins, and they contribute to specific evolved biological processes. It then concludes by applying the notion to two case-studies, haemoglobin and crystallins, and by evaluating the view according to criteria for functions.

## Where is the problem with proteins functions?

The difficulty in determining protein function, alongside relevant empirical considerations, arises from the complex nature of protein. They are chemically defined as polymers of amino acids. They are evolutionary defined as biomolecules evolved from nucleotides and the result of the expression of selected genetic sequences. In biological systems, they are defined by the multiple functions they display. These dimensions are widely regarded as interconnected. Thus, if we want to explore the function of proteins, we need to take into account the different structural properties and the different ways in which proteins’ functions can be characterised. This can be unpacked into two problems, which can also be applied to the function debate more in general. The first (i) is the function bearer question, asking what we ascribe functions to (traits, molecules, parts of traits etc.), the second (ii) is the function concept question, asking which notion of function is best applicable to the answer to (i).^4^ In the context of proteins, these can be framed as:

- The structure problem (2.1): which proteins’ structural properties are relevant for their functions?
- The function problem (2.2): which notion of function captures proteins’ functions?

### The structure problem

**Fig. 1:**
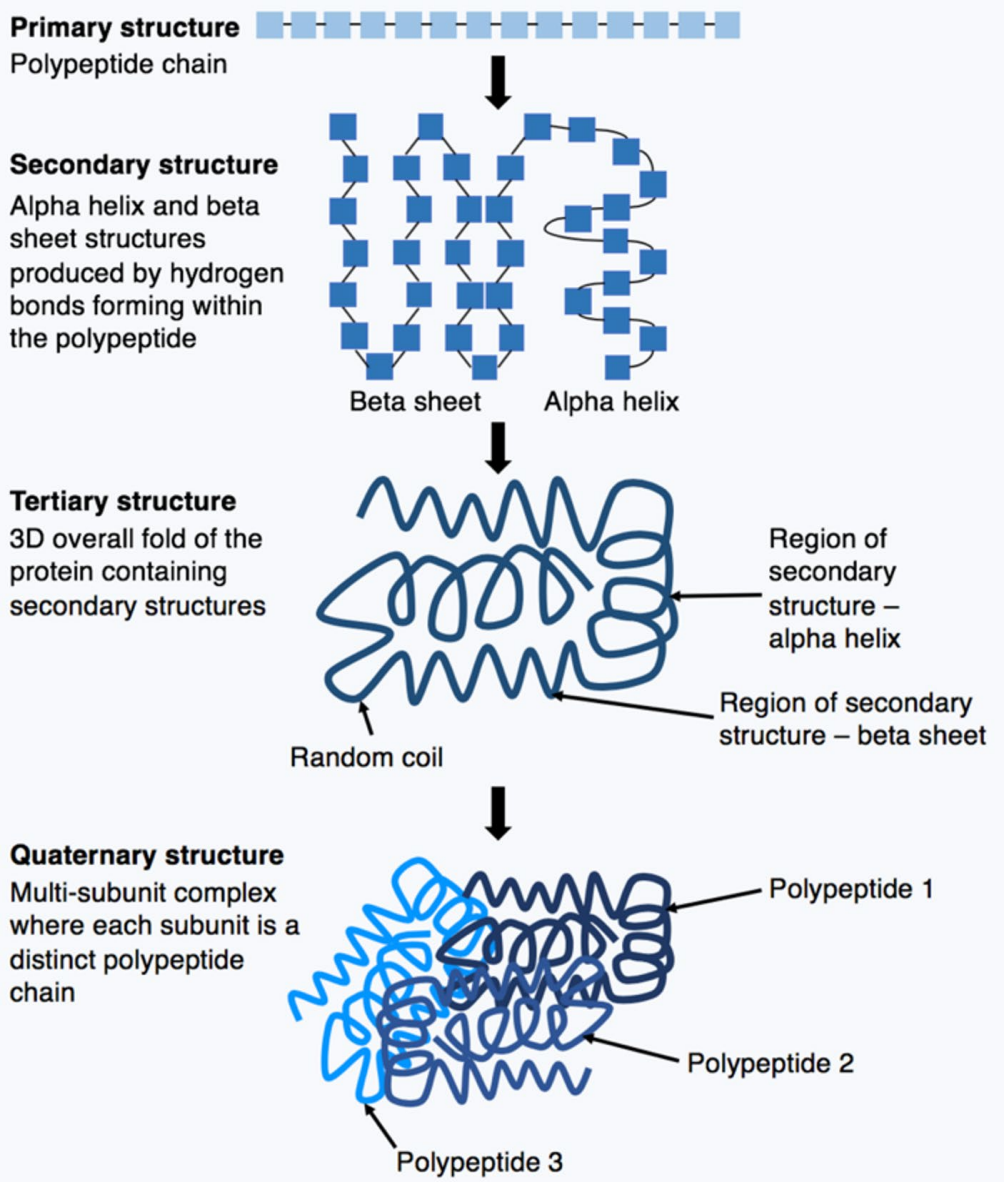
Illustration of protein folding highlighting the four stages of protein structure. It is possible to see the primary structure, the secondary structure and the folded tertiary and quaternary structures. (Kep17, CC BY-SA 4.0 < https://creativecommons.org/licenses/by-sa/4.0 > , via Wikimedia Commons. https://commons.wikimedia.org/wiki/File:Protein_folding_figure.png )

One of the goals of biochemistry and structural biology is to correlate structure with function, so it is important to consider which aspects of structure are relevant for functionality. Proteins present a three (or four) levels structure, each of which is characterised by different relevant properties (Fig. 1). Proteins have a primary structure characterised as a linear sequence of amino acids, linked mostly by covalent bonds (peptides). The secondary structure is given by the folding of the primary structure into stable geometrical patterns, depending on specific properties of the amino acids, and is exemplified by alpha helix and beta sheets. It forms due to localised hydrogen bonds between the amino hydrogen and carboxyl oxygen atoms in the peptide backbone. While most proteins present a secondary structure, some have areas that don’t. The tertiary structure rises when the polypeptide takes a complex three-dimensional molecular shape, generally caused by side-chain interactions such as ionic and hydrogen bonds, disulphide bridges, and hydrophobic and hydrophilic interactions. Finally, some proteins reach a quaternary structure given by the aggregation of folded protein chains. The final stable folded structure able to perform a function is called native state or native structure.

Understanding how and why folding happens in the way it does is often referred to as the problem of protein folding.^5^ This is because the primary structure could fold in many ways, some of which are not relevant for the given protein function, and it is a phenomenon heavily dependent on context and presence of helper proteins (*chaperonins*) (Slater 2009; Santos et al. 2020).

The structure problem concerns which of these levels of structure is the function bearer and the current debate can be summarised around whether the primary structure is sufficiently linked to the protein’s functions. On the one hand, Slater (2009), Tobin (2010) and Santos et al. (2020) argued against the sufficiency of primary structure as ‘‘[a]n amino acid sequence alone has no direct link with a protein’s ultimate biological role’’ (Slater 2009,852). On the other hand, Goodwin (2011) and Tahko (2020) argued that the primary structure of the protein is the most fundamental property characterising functionality. This argument is based on stability, as primary structure remains “stable”, and reducibility, as the properties of the primary structure determine the subsequent stages in folding. The relation between structure and function is complex due to the presence of multiple realisability and multiple determinability (Slater 2009; Tobin 2010; Tahko 2020; Bellazzi 2023).

Multiple realisability is the possibility that the same tertiary and quaternary structure and the same kind of protein can be realised by different chains of amino acids.^6^ One of these cases is haemoglobin. Haemoglobin is a protein of red blood cells with the function of binding and releasing oxygen in blood. This protein can be realised by the folding of at least six known different globin polypeptide chains. Moreover, haemoglobin presents an prosthetic heme group, which is crucial to display its function of binding and releasing oxygen, and it can be discussed how and whether this group is part of which structures or whether it is an extra structural feature.^7^ This makes the direct relation between structural properties and functional ones problematic, especially if the focus for proteins’ functionality is on the primary structure.

Multiple determinability can happen in (at least) two ways (Tahko 2020). Firstly, some amino acid chains can be folded in different tertiary and quaternary structures, as in the case of intrinsically disordered proteins presenting different functions due to having a highly flexible polypeptide chain. Secondly, the same folded structure can have different functions because its parts can perform differently in various environments due to their conformation. This is the case of moonlighting proteins, such as crystallins. These play a function in sight and one in digestion performing in different contexts (Tobin 2010; Bartol 2016). Multiple determinability challenges the possibility of a 1:1 relation between structure and function, as the molecular environment of the protein has a core role in determining the folding process and the realised function (Slater 2009; Santos et al. 2020). The complexity of proteins’ structure generates the first challenge regarding proteins’ functionality.

### The function problem

The function problem asks which accounts of functions are relevant for proteins and their different properties (see Ratti and Germain 2022; Cumisano, Sterner 2019). The literature on biological functions has been growing extensively in recent years and comprises many different accounts. There are two main accounts of biological functionality that have been applied explicitly to molecular cases: the causal theory of function (Cummins 1975, Craver 2001) and the evolutionary theory of function (Slater 2009; Bartol 2016; Tahko 2020; Bellazzi 2025).^8^ Together with these, I have suggested a *sui generis* account of biochemical functions (Bellazzi 2025). For the sake of simplicity, this paper focuses on these three accounts of functions as they have been explicitly applied to molecular case studies. Sect. "Shape is everything" will also discuss how the suggested view can be compatible with the organizational view of function (Saborido et al. 2011), while not entering into the details of this latter account here.

The causal theory of function defines a function as the causal role of an entity within a performed activity in a complex system (formulated originally by Cummins 1975). This theory works well for molecular case studies and for *chemical functions*, as it is possible to identify the causal function of a molecule by identifying its specifical structural features and functional groups (Dupin 2023; Bellazzi 2025). Chemical functions are defined in relation to the functional groups within the molecule that allow contribution to given reactions. For instance, ascorbic acid has an acidic functional profile because it has a hydroxyl group. In this case, the focus is on structural properties, specifically functional groups, that in the case of the protein can be ascribed to the primary structure (as in Goodwin 2011; Tahko 2020).

According to an evolutionary view, the function of a trait is determined by that trait’s evolutionary history (as for instance in Millikan 1989; Neander 1991; Garson 2019 Fagerberg and Garson 2024). Specifically, according to selected effect theory (one of the most prominent in ascribing evolutionary functions) a trait’s function is a difference-maker effect of such trait that contributed to the fitness of the ancestors and has been retained via natural selection because of these benefits (see Christie et al. 2022). For example, the heart has a function of pumping blood because it contributed to the inclusive fitness of those having a pumping blood heart. The proteins’ function could then be determined by the proteins’ evolutionary history (as in Bartol 2016) and by which amino acid sequences have been evolutionarily retained in relation to the given genetic sequences. In this case, the function is not determined as much by structural considerations in themselves, but by the evolutionary selection of given genetic sequences encoding for specific amino acids chains, forming the primary structure of the protein.

As a middle ground between these two, I provided a definition of biochemical functions that brings together insights from the causal role and the selected effect views (Bellazzi 2025). Biochemical functions are associated with a set of chemical dispositional properties to bring out a specific effect within biological processes and the relevant chemical dispositional properties are (at least) indirectly evolutionary selected (2025). In this case, the function of vitamin B12 to contribute to erythropoiesis is identified thanks to those functional groups that contribute to the evolutionary selected process of erythropoiesis. The account does not really represent an alternative to the causal theory or the selected effect theory given that it is not generalisable beyond biochemical cases. However, it is a compromise between the two as brings together the evolutionary context within a chemical view of functions, allowing to identify the relevant causal contributions for the considered process. This view also considers the environment in which the function is played, allowing for causal role of the cellular, organismal and environmental relations in protein development (in line with Slater 2009; Santos et al. 2020). It is important to notice that the main focus of this account is on the chemical functional groups (*chemical functions)* of the primary and secondary structure that allow for the given effect in the evolutionary selected context. This view will be the starting point for the present analysis of proteins’ function (Sect. "Shape is everything").

## Shape matters for function

The first step is then to answer the structure problem. Some authors, as Goodwin (2011) and Tahko (2020), suggested that proteins’ functions can be reduced to the primary structure and secondary structure of proteins. Others, as Slater (2009), Tobin (2010), Bartol (2016) and Bellazzi (2023), consider functions as the main property of proteins, but do not present a specific account of proteins’ function. In this section, I will explore why primary and secondary structure are not the right properties for proteins’ functions (as also Santos et al. 2020). Instead, I will argue that we should focus on tertiary and quaternary structure.

One of the main problems of considering primary and secondary structure for proteins’ functions is the aforementioned presence of multiple realisability and multiple determinability: different amino acid chains can realise the same protein with the same functional profile and the same amino acid chains can be determined into proteins with different functional profiles or even objects not characterised as proteins (such as bioplastics). To illustrate this point, let us consider again haemoglobin. Haemoglobin has the function of binding and releasing oxygen in red blood cells and it can be composed of different amino-acid chains which can then fold into the same tertiary and quaternary structure, for instance in humans there are 6 types of chains.^9^ In this case, each primary structure (the amino acid chains considered) and each secondary structure (how the amino acid chains form stable geometrical patterns) are not specific enough for haemoglobin’s function as there could be others that realise the function. This makes the function multiply realised. Moreover, there may be alternative configurations beyond the one that realises the relevant function, making the primary and secondary structure possibly multiply determinable. This is because the process of protein folding comprises a complex set of interactions, such that the tertiary and quaternary structures are not the only conformations accessible to a given primary sequence.^10^ This suggests that the primary and secondary structures lack the kind of stability and fundamentality ascribed to them (in Goodwin 2011), insofar as the properties of the primary structure and secondary do not yield univocally the subsequent stages via strong chemical bonds, nor the functional profile associated with them. Although the primary and secondary structures are components of the folding process, their properties alone do not suffice to make them function bearers, as they lack the requisite specificity for functional individuation.

This leads us to consider the tertiary and quaternary structures, which are scientifically widely recognised as those relevant for a protein’s function. As aforementioned, these structures cannot be easily reduced to the primary or secondary structure.^11^ The final three-dimensional conformation reached by the protein, commonly referred to as its *native state*, is the “shape” that underpins protein functionality. While the specific characterisation of each native state depends on the individual protein under consideration, generally it corresponds to its tertiary or quaternary structure with the minimum free-energy. However, this does not imply that the final conformation has a fixed geometry. Rather, it is better described as “a combination of several conformational substates that collectively constitute the native state ensemble” (Mishra and Jha 2022, p. 10). While it is possible to identify a protein native state, each state presents a degree of conformational heterogeneity that is necessary for protein’s functionality. Thus, focusing on the final three-dimensional shape does not entail a static view of protein structure while acknowledging the relevance of geometrical aspects for functions. Moreover, a protein can retain its native state, with a completed folding process, even though parts of its structure may shift in response to environmental and functional changes (Gupta and Uversky 2023). The precise identification of which components of the proteins’ native structure are relevant for functions is still discussed in the scientific literature (Mishra and Jha 2022). Nevertheless, the function-bearer in proteins can be located in the folded structure, which may also include non-amino acid components (such as prosthetic groups) in cases where these are required for the protein to become functional, as in conformational proteins. Let me support this further by considering haemoglobin again.

In haemoglobin, the native state, which comprises the tertiary and quaternary structures and the heme group, enables the protein to perform its function of loading and unloading oxygen. These structures adopt conformations that allow the protein to perform the function in a more efficient and responsive way. This is because the various subunits of haemoglobin bind and release oxygen in a way that modifies the quaternary structure, facilitating oxygen binding by other subunits (Szczesny-Malysiak et al. 2020). Moreover, the quaternary structure provides binding sites for allosteric regulators, such as 2,3-bisphosphoglycerate (2,3-BPG), which modulate the molecule’s affinity for oxygen under different conditions. This suggests that the relevant structure for protein function is the native structure, encompassing the tertiary and quaternary structures along with associated relevant functional groups and the heme group. This is further supported by cases in which haemoglobin is composed of different amino acid chains but still attains sufficiently similar tertiary and quaternary structures, allowing for the function of oxygen binding and release to be realised optimally (Thomas and Lumb 2012).

Let’s consider the case of crystallins too (as in Tobin 2010; Goodwin 2011; Bartol 2016). In most mammals, crystallins are able to play an enzymatic function in digestion and a structural function in the crystalline of the eyes. These proteins are able to play different functions by having a generally fixed folded structure and are often classified as the same protein. This can be a challenge to this answer to the structure problem, as their native structure remains mostly invariant in these cases and there is no one-to-one correlation between the folded structure and function (as in Goodwin 2011). However, a closer examination of how different functions are achieved reveals the importance of protein shape. Specifically, the final folded state is not a single fixed structure but rather a collection of possible conformations enabling the protein to interact with various components in different environmental and cellular contexts (Santos et al. 2020). Crystallins adopt a compact, stable form when functioning in sight, but shift to a more dynamic configuration when performing enzymatic functions. The protein doesn’t unfolds or refolds, but, rather, it undergoes a transition between a more structural configuration and a more dynamic one that facilitates its catalytic activity (Gupta and Uversky 2023). While the shape of the protein remains fixed, it is the fact that the protein has *that given native* structure which allows for its multiple determinability and the possibility to display different functions in different contexts.^12^ In this case, the different functions of the protein arise from the interaction between the molecular environment and the protein folded structure.^13^ Protein shape matters for functionality. The next step is to explore which accounts of function best captures this.

## Unpacking the function problem

In this section, I will consider the function problem and theories of function in light of the answer to the structure problem. This does not come as a criticism to the views in themselves but rather to their application to the protein case.

As aforementioned, the causal theory of function (formulated originally by Cummins 1975), defines function as the causal role of an entity within a performed activity in a complex system. This theory works well for molecular case studies and for *chemical functions* (Dupin 2023; Bellazzi 2025). However, while a chemical view of functions can be considered informative for proteins’ functionality, the causal contribution account is not specific enough for biochemical functions. A chemical view doesn’t disentangle causal contributions or contributions to chemical reactions from biochemical functional ones (Bellazzi 2025). For instance, the function of haemoglobin of carrying oxygen is more specific than the chemical functional profile of haemoglobin, which presents a long chain of amino acids with different functional groups. Moreover, haemoglobin (as other proteins) is realised by different amino-acids chains, which can present different functional groups and different properties, and this multiple realisability represents a challenge also to a chemical view of function. This problem is also valid within the physiological context considered, as we can imagine that (chemically) haemoglobin could react in ways that do not involve oxygen binding. Lastly, a chemical view of function does not explicitly consider the biological context in which the proteins is functional: any chemical context in which the functional group can react would become a possible context for the manifestation of the proteins’ functionality.^14^

What about a selected effect view of function in line with giving priority to evolutionary features? The application of a biological account of function to molecular cases encounters two main problems, a trait individuation problem and a function ascription problem (Bellazzi 2025).^15^ The trait individuation problem regards the fact that it is difficult to conceptualise proteins as “traits” bearing functions, as these are generally defined as detectable phenotypic properties of organisms or phenotypic contributions to fitness or structural features. Proteins are instead chemical macromolecules, and thus different from traits. Moreover, individuating traits is a problem in itself (as analysed by DiFrisco and Ramsey 2023).^16^ The second problem concerns the fact that a selected effect view of functions is suited to account for the whole proteins’ synthesis process as a target of selection, rather than proteins’ functions in themselves. To illustrate the difference, the question does not ask what the function of the process synthetising haemoglobin is, but rather what the function of haemoglobin is and how should we characterise it as such. This pertains the molecule and not the correlated genetic sequence. Having said that, a version of the selected effect theory could be amended to include selected genetic sequences, as well as traits, as function bearers, including genotypes and then proteins whose activities contribute to the expression of the relevant phenotype.^17^ In this case, proteins’ functions could be reduced to the function determined by the evolutionary selection of given genetic sequences encoding for the specific amino acids chains of the primary structure. This provides two routes for proteins’ functionality. The first one is to ascribe some proteins a form of biological evolutionary function, identified in terms of the retained genetic sequence. However, this offers us a link to the primary structure (the one more directly encoded by the relevant genetic sequences), but not a direct one to the tertiary and quaternary structure (at least for some proteins) given the complexity of protein folding and the aforementioned problems correlated to ascribing functions directly to the primary sequence. The second route is to see the role that evolution plays in function ascription to protein thanks to the process of protein synthesis or in the context in which the protein exercises its function, while the main function bearer is still the final folded structure.^18^

As mentioned above, both the causal contribution and an evolutionary view of functions have merits and shortcomings in themselves. However, here the focus is on the problems regarding their application to biochemical and molecular cases, and for which these two views cannot be applied as such. I have dealt with some of these problems by formulating a specific account for biochemical cases (Bellazzi 2025). While this proposal might be suited for proteins, it still presents some shortcomings as it focuses on properties ascribable to the primary and secondary structure and does not consider the relevance of the folded structure. This is because functional groups or “relevant chemical dispositional properties” are properties of the primary and the secondary structure, and while they can be relevant for proteins’ functionality they are not sufficient (as previously analysed). Let us consider haemoglobin again and the function to bind and release oxygen responsively (we will go back to this in detail in §5.1). The presence of the “heme” group is crucial for this function, as an heme group is a porphyrin ring with an iron atom attached necessary for the binding of the oxygen, as captured the biochemical account. This group has to be embedded into the final structure of haemoglobin. However, it is the tertiary and the quaternary structure which allows for the responsiveness and the needed release of oxygen at the right moment. These features are not captured by the account: the biochemical view of function does not consider the relevance of the geometrical and physical properties of the molecule (at least not explicitly). This makes the account a good starting point to discuss proteins’ functions, but it has to be modified to be consistent with the answer to the structure problem.

## Shape is everything

This section focuses on the function problem by building on the biochemical account of functions and considers the geometrical and physical properties of the folded structure as the relevant ones. As mentioned above, biochemical functions can be defined as functions associated with a set of chemical dispositional properties to bring out a specific effect within biological processes and the relevant chemical dispositional properties are (at least) indirectly evolutionary selected (Bellazzi 2025). This account is lacking the reference to geometrical structural properties, crucial in the answer to the structure question, and focuses mostly on functional groups. Thus, it can be modified as follows:

*Proteins’ functions*: are *(i*) dispositions associated to *(ii)* specific chemical and physical properties of the proteins’ folded structure (the final native state of the protein) that allow for specific binding sites.^19^. The specificity of the properties of the native state relevant for different functions is *(iii)* determined by the evolutionary selected processes to which the proteins contribute.

Let us unpack the main components of this proposal. Firstly, *(i)* proteins functions are dispositional properties. This is in line with my first proposal for which biochemical functions are dispositions that manifest specifically in given circumstances (Bellazzi 2025) and with a recent account put forward by Hundertmark and Bos (2024).^20^ The function of the protein is a complex disposition present thanks to the shape of the protein and that contributes to the considered biological processes, and not any causal chemical disposition (differently from a chemical view of functions). Considering the function of the protein as a (complex) disposition determined by the shape has a series of advantages. Firstly, it allows the protein to respond to the environment in different ways, allowing for response functions (Hundertmark and Bos 2024). For instance, haemoglobin’s function to carry oxygen is a response dependent disposition as haemoglobin has the disposition to be more or less affine to oxygen in different conditions thanks to the native structure which allows for binding sites for allosteric regulators. Secondly, some dispositions can be seen as multi-track, manifesting in different ways depending on the circumstances that trigger their manifestation (Hundertmark and Bos 2024). This allows proteins to display multiple functions while maintaining the same native structure or using specific chemical and physical properties to realise different functions. This is consistent with moonlighting or the case of intrinsically disordered proteins.

The second feature (*ii)* of the account is that it focuses on the shape of the folded protein, associating the function with the relevant chemical and physical properties of the proteins’ native structure and the specific binding sites. This combines geometrical and chemical properties: the shape is a core feature of the protein to realise the function in relation to the structure problem and needs to present the relevant functional groups and binding areas to allow for the realisation of the function. Moreover, the final folded state is a combination of the possible different configurations reached by the protein that allow for the proteins’ function. Accordingly, the account extends the biochemical function proposal by considering both the relevant functional groups and the properties of the folded native state of the protein. For future development, it is worth noticing that focusing on dispositions of the structure can allow to consider the overall relevance of shape and morphology also for the functionality of other traits and at higher levels.

The third feature (*iii*) of the account allows to determine proteins’ functions and their specificity. Here, I argue that such specificity is determined by the evolutionary history of the process to which the proteins contribute (as in Bellazzi 2025). For instance, crystallins’ function in sight is so because “eyes with crystallins” have been selected. This allows for the specificity of the disposition, as the given biological process or trait presenting the protein has been selected to trigger the manifestation of the relevant protein’s disposition. This sets proteins’ functionality in an evolutionary context, being consistent with accounts of selected effect views of biological functionality. The account is thus *specific* without incurring into the problem of discipline-relativity discussed by Tahko (2020). According to this criticism, a dispositional view of protein’s functions implies that the function of the protein is determined by the discipline studying the given protein and thus does not have a real ground or profile (in comparison to the more stable primary structure). However, it is the evolutionary history of the context which determines the function specificity and not the discipline. This also gives the advantages of historical views of functions to this proposal, as analysed extensively by Garson (2019, 2023) and Christie et al. (2022).^21^ Such characterisation of proteins’ functions allows proteins to also bear selected-effect evolutionary functions, in the case in which the sequence encoding for the proteins’ primary structure has a clear evolutionary history linked to the function of the protein, as mentioned in Sect. "The function problem". In this case, the protein presents a “dual function”, a more directly evolutionary one, with the primary sequence as function bearer and a biochemical one, with the folded structure as the function bearer.

Lasty, this account can in principle be compatible with other views of functions that generally consider higher level features, such as the organisational account (OA), which is framed within system biology and for whom the integration of the chemical and biological features of organisms is crucial (Mossio et al. 2009). While the literature on biochemical kinds considered here has not applied this view of functions to molecular cases, the proposed view of proteins’ functions is compatible with this framework. According to the OA, a trait type T has a function if, and only if, it is subject to organizational closure C in a differentiated self-maintaining system S (Mossio et al. 2009, 828). Specifically, once we assume that functions can also be ascribed to non-traits, we could see a given protein having a function because it is subject to the organisational closure of its folding process, where the final native state configurations could be seen as constraints. Moreover, this configuration contributes causally to the given organism that represent the self-maintaining system of reference, allowing for the difference levels of interdependence among different components of the biological system. This contribution can be seen as indirectly evolutionary selected. Thus, the presented view of functionality can also fit within the OA.^22^

In conclusion, structure and function are not in contrast, but two sides of the same coin: structure bears and constrains function, while functions maintain their specific causal profile in the right context. This also allows, against Bartol (2016), to have a unified presentation of proteins for which there is no need to contrast chemical kindhood, characterised by structure, and the biological kindhood, characterised by function, as the two are deeply related.

### Haemoglobin and crystallins: case studies

To illustrate the account, let me consider more in detail the two main case studies mentioned throughout the paper, haemoglobin and crystallins (as also in Tobin 2010; Goodwin 2011; Bartol 2016; Tahko 2020). Haemoglobin’s function is the one to load and unload oxygen effectively. In the proposed framework, the function is the complex disposition associated native state of the protein, comprising the heme group, responsible for oxygen binding, and the properties of a plastic quaternary structure that allows the protein to be responsive in its function and allows the binding sites for allosteric regulators, as 2,3-BPG, and this makes the molecule be more or less affine to oxygen in different conditions (Thomas and Lumb 2012). This modularity in function manifestation is captured by the dispositional nature of the function. Moreover, the focus on the shape of the protein and on those chemical-physical properties allowing for the relevant bonds is compatible with multiple realisability, given the presence of different amino acid chains that fold in haemoglobin proteins. The folded haemoglobins present different components, while maintaining the same shape and the relevant chemical properties, such as the presence of a heme group, to realise the function. The specificity of these properties and the presence of a given shape and a heme group is given by the evolutionary history of erythroblasts and generally of the process of blood circulation, as it is such context that determined—evolutionary—which properties of haemoglobin are relevant rather than others.

A similar analysis can be provided for crystallins, globular proteins that have a structural function in sight and an enzymatic one in digestion. The protein has at least two functions, which are dispositions associated to specific chemical and physical properties, comprising the relevant functional groups and the proteins’ folded state (the shape of the protein).^23^ This is a case of multiple determinability, as the same native structure can serve various functions by adapting its structure to different environments based on compatible configurations. These cases might be considered a challenge to the account, as the folded structure might be seen as not “specific enough” to identify the function of the protein, given the lack of one-to-one correlation. This challenge can be addressed by noting that crystallins present different functions *because* various parts of their structure interact differently across environments.^24^ This allows the identification of the structural properties relevant for the given functions and how these parts react differently in the two molecular environments (Tobin 2010; Goodwin 2011). Moreover, it is the different evolutionary history of sight and of digestive processes that identifies which properties are relevant to realise the different functions (in line with the account suggested). This is consistent with the analysis of moonlighting proteins for which the multiple functionalities are expressed by considering the evolutionary tree of both the process and the gene sequences related to the protein and by considering the different biochemical pathways (Jeffrey 2014; Boniolo and Campaner 2018). Lastly, the lack of one-to-one correlation between the folded structure of a protein and its biochemical function should not be a challenge in general to the account. As underlined by Havstad (2018), it is not rare in the chemical world (nor in general) that one entity can perform more than one function, as in the case of crystallins.

### Are these functions?

This account presents many advantages which allow us to answer to the function problem. In this subsection, I will go through the different features relevant for accounts of functions. Specifically, (i) the account captures the specificity of proteins functions and is consistent with the answer to the structure problem; ii) the accounts distinguishes functions from mere effects; iii) the account is able to deal with dysfunction, that is normativity (Garson 2019).

Firstly, this proposal focuses on the relevant properties for proteins’ functionality by associating functions with specific properties linked to the shape of the protein, in line with the answer to the structure problem (i). Secondly, functions are different from mere effect because they refer to selected dispositions, either directly or indirectly (Hundertmark and Bos 2024). For instance, the function of haemoglobin is the disposition to bind and release oxygen appropriately and not any chemical reaction in which the protein can enter due to the presence of the heme group (ii). This is so because the evolutionary history of the process to which the molecule contribute allows to identify the relevant function, bearing the advantages of evolutionary accounts. Moreover, the specificity of protein’s function arises from the account’s ability to integrate features of structure, evolutionary history, and environment. Considering the proteins’ native structure shows how proteins’ functions is dependent on the chemical components and structure of the molecule. The sequence of amino acid can also display direct evolutionary selection and history, and this can be reflected in the proteins functionality. The dispositional nature of proteins functions allows for context-relative considerations regarding the manifestation of the relevant function, as in the case of moonlighting proteins.

Lastly, accounts of functions need to be able to deal with dysfunctions (iii). Dysfunctions are considered as such if there is something in the constitution of the function bearer for which it is not able to perform its function (Bellazzi 2025; Garson 2023 Hundertmark and Bos 2024). In the case of proteins, there are two main ways in which they can be dysfunctioning, illustrated here with the case of sickle cell anaemia (also in Tahko 2020). In this disease, the replacement of a single amino acid in two of the haemoglobin’s four amino acid chains (glutamic acid replaced by valine*)* alters the molecular surface of the protein that causes aggregation upon deoxygenation. This change in the protein’s structure has an impact on the shape of the erythroblast cell, which then presents less flexibility, and can lead to the typical “crescent moon shape” characterising some of the erythroblasts in this disease (Christop et al. 2005). Firstly, we can say that protein is dysfunctioning because the protein folding process didn’t occur appropriately. The protein does not present the native structure relevant for its functionality and this reduces the capacity to bind and release oxygen. In the case of sickle cell anaemia, this is caused by a gene mutation. Extending this notion of dysfunctionality, there can be cases of misfolded proteins due to alterations in the environment and which can lead to a series of diseases. Secondly, proteins can be dysfunctioning if there is a mismatch between the historical evolutionary selection of a given shape and the environment in which the protein plays its role. While sickle cell anaemia is mostly considered a disease, the gene encoding for this mutation has been evolutionary retained in contexts in which having such a difference in the protein’s shape has been advantageous in the past, as in environments in which malaria is well spread. The presence of cells less affine to oxygen increases the concentration of CO_2_ in the organism and this contrasts the proliferation of the plasmodium causing malaria. This allowed for a better survival of those bearing the mutation and then the transmission of the relevant gene to offsprings. In current times, the protein is dysfunctioning not (only) because there is a change in its shape, but also because the selection of such shape is not helpful anymore in those environments in which malaria is absent or there are alternative treatments.^25^ The consideration of these cases allows to illustrate an interesting difference between malfunctioning and dysfunctioning. A variation in quantity of a given protein, either by absence or by excess, can lead to a malfunction (rather than dysfunctioning), in the case of haemoglobin a malfunction in carrying oxygen. This is identified by the evolutionary history of the relevant process and its alterations. Differently, a misfolded or a differently folded protein can be said to be dysfunctioning because there is an alternation in the chemical and physical properties associated to the function.

## Conclusion

One of the main questions for biochemistry regards the relation between structure and function for proteins. This has also been acknowledged in the philosophical debate, such as Slater (2009), Tobin (2010), Goodwin (2011), Bartol (2016), Tahko (2020), Bellazzi (2025). This paper builds on the discussion initiated by these works to analyse the question of how to attribute functions to proteins and argues for the thesis that “shape is everything”. Proteins’ functions are associated to dispositions present thanks to specific chemic-physical properties of the molecule in its native structure, comprising the tertiary and quaternary structure and functional relevant features. These specific properties are identified by looking at the evolutionary history of the relevant processes. The main contribution of this account is that it addresses protein shapes as the explicit bearers of functions for proteins. I have done so by unpacking two problems, the structure problem, which asks which structural properties are relevant for proteins’ functionality; and the function problem, which considers which account of functions is relevant for proteins’ function. The answer to the first is offered by proteins’ native structure, in line with scientific research. The answer to the second is given by a view of functions that considers the geometrical properties of the protein, expanding the original characterisation I presented in Bellazzi (2025).

First, this paper contributes to the broader biological function literature by highlighting explicitly the role that structures play for biological functions more in general and by bringing together advantages of three main accounts of functions, the causal view of function, the selected effect view of function, and of the recent analysis of biochemical functions offered by Bellazzi (2025). This also allows the account to be compatible with other views of functions, such as the organisational view of functions. Second, this paper can contribute to the debates on the differences or similarities between chemical and biological entities. It presents a view of proteins’ functionality that can offer a more unified picture given that structure and function can be considered two sides of the same coin. Thirdly, it allows for a better understanding of the kindhood and classification of proteins and contribute to the debate between pluralism (Slater 2009), functionalism (Tobin 2010; Bellazzi 2025), reductionism (Goodwin 2011; Tahko 2020), and dualism (Bartol 2016) regarding biochemical kinds.

## Footnotes

1 Among many Nobel Prizes on protein structure and function, the Nobel Prize in Chemistry 2024 is the latest. This prize awarded AI tools able to predict protein structure and laboratory work on artificial synthetic proteins.

2 This is a broad question discussed in molecular biology, structural biology and biochemistry and within philosophy of biology and by recent philosophical literature like Slater 2009; Tobin 2010; Goodwin 2011; Germain et al. 2014; Bartol 2016; Havstad 2021; Tahko 2020; Bellazzi 2025; Ratti and Germain 2022. The debates on proteins’ function also involve discussions on the central dogma and on the relationship between amino acid sequences and the folded structure, as in Morange 2006; Mosini 2013.

3 In this text, I use function and functionality as synonyms, given that functions are understood dispositionally (as analysed in Sect. "Shape is everything").

4 I thank reviewer 2 for framing the problems in these terms and making this suggestion.

5 The answer to the problem of protein folding is tackled empirically using both in vitro and in silico methods, such as AlphaFold.

6 A further form of multiple realisability can be seen in the various “alleloforms” displayed by various proteins, forms in which very slightly different polypeptide sequences maintain the protein’s tertiary structure and function, as for instance the alcohol dehydrogenase (ADH) enzymes in *Drosophila*. These enzymes have a function in alcohol processing and they occur in two forms, one named “slower” and one named “faster” as they display the same function in different speeds (example from Slater 2009).

7 I thank reviewer 2 for pointing this out.

8 The literature on biological functions presents different accounts. Some are the organisational view (Saborido et al. 2011), the goal-contribution view, the forward-looking dispositional view (Bigelow, Pargetter 1987) and mechanistic organisational views (Maley, Piccinini 2017). Apart from the forward-looking dispositional view of function, whose shortcomings are analysed in Bellazzi (2025), the other views are generally proposed for more complex traits or systems and for entities that are not molecules. A more detailed analysis could be provided, but it would extend beyond the scope of the paper and the author has decided to focus on the accounts that have been applied to biochemical kinds.

9 For the sake of simplicity, I am considering carrying oxygen in a responsive way as the main function of haemoglobin as what can be considered the proximal function of the protein. This does not imply that this should be the only function of the protein as it is possible to account for others depending on ones’ view of proximal and distal functions (see Fagerberg, Garson 2024). The account remains neutral regarding whether only proximal functions are proper functions.

10 Protein-folding is often subsumed under self-assembly and considered a paradigmatic case of biological complexity due to the process tending towards energy minimization processes and thermodynamic equilibrium by using hydrogen bonds and other weak bonds and interactions (Vendruscolo et al. 2003; Halley, Winkler 2008).

11 Slater (2009, 852) writes that a protein must be “folded” to acquire their function, also Tobin (2010) underlines the relation between structure and functionality. This is also supported by the multiple research projects linking functionality with the tertiary and quaternary structure of proteins.

12 Proteins’ function can change with conformation, but whether we take this to mean that protein should be classified according to its primary structure or its function is a different question (discussed in Slater 2009, Tobin 2010, Goodwin 2011, Havstad 2018). Mishra and Jha also underlines how flexibility and dynamics are the two main strategies allowing proteins to perform complex functions at reduced costs. Moreover, while there is an overall agreement on the fact that proteins’ function depends on the final folded structure, which parts are exactly constituent of the protein native structure and the determination of functionality is still under empirical research (2022).

13 Intrinsically structurally disordered moonlighting proteins that present different functions due to having a highly flexible polypeptide chain could be a possible counter example. In this case, these proteins present different structures according to cellular conditions and contexts and the function they have to perform. Thus, the while the link between protein and tertiary/quaternary structure is challenged, the link between structure and function remains available. For more on intrinsically disordered proteins see Tobin 2010.

14 As Neander (1983) pointed out, Cummins’ theory tends to define the relevant context in terms of the relevant discipline interest. This way to pick out context is subject to the criticism of Tahko (2020) that the identification of functions is simply a matter of discipline relativity and thus we should instead focus on primary structure as something stable. I will consider this objection in more detail later in Sect. "Shape is everything".

15 The selected effect view of functions encounters also other problems, such as being a reminiscent of teleological talk being unable to deal with novel mutations and artifact functions, being inconsistent with scientific research (Maley and Piccinini, 2017; Ratti and Germain 2022; Saborido et al. 2011). However, these problems regard the theory of functions in general, rather than their application to biochemical and molecular cases, and thus will not be addressed here as they go beyond the scope of the paper.

16 This problem can be generalised to other accounts of functions that ascribe functions to traits.

17 I thank reviewer 1 for pointing out this aspect.

18 This tension, also mentioned in 2.2, can be dealt with by allowing proteins to display both evolutionary selected functions and specific proteins’ functions, or biochemical functions, as will be explored in Sect. "Shape is everything".

19 Specific binding sites can be identified as regions on a protein's surface that allow the protein to interact with other molecules via different chemical bonds, the binding partner are generally referred as ligands. There are different binding sites and generally the ones relevant for functionality are called “active sites”, however also other types like allosteric sites or multi-molecules ones. The relevance of binding sites for functions in evolutionary context has for instance been analysed in Abrusán and Marsh (2018) in the paper “Ligand Binding Site Structure Influences the Evolution of Protein Complex Function and Topology”.

20 The reference to biochemical functions as dispositions or “biochemical dispositions” is also present in Slater (2009) and discussed in Tahko (2020).

21 Despite its focus on evolution, the account can also be compatible with views identifying the specificity of the function by looking at a organismal systems which allows for the identification of the contribution in a way which is not ambiguous. A similar position is discussed in Boniolo and Campaner (2018) where they argue that pathways specify how molecular context constrain the performance of the function.

22 More could be said on this. However, it would be beyond the scope and the limit of a single paper. I thank the reviewer for this suggestion.

23 Recent research has underlined that many proteins display multiple functions (Mishra and Jha 2022).

24 It is important to underline that some moonlighting proteins can perform their functions thanks to different part of their globular structure simultaneously, while others change their function in response to changes in the environment (Jeffrey 2014).

25 The works of Justin Garson in relation to how selected functions can become mismatched with the environment is also relevant.
